# Epithelial to Mesenchymal Transition by TGFβ-1 Induction Increases Stemness Characteristics in Primary Non Small Cell Lung Cancer Cell Line

**DOI:** 10.1371/journal.pone.0021548

**Published:** 2011-06-30

**Authors:** Giuseppe Pirozzi, Virginia Tirino, Rosa Camerlingo, Renato Franco, Aantonello La Rocca, Eleonora Liguori, Nicola Martucci, Francesca Paino, Nicola Normanno, Gaetano Rocco

**Affiliations:** 1 Department of Experimental Oncology, National Cancer Institute, Naples, Italy; 2 Department of Experimental Medicine, Second University of Naples, Naples, Italy; 3 Department of Pathology, National Cancer Institute, Naples, Italy; 4 Department of Thoracic Surgery and Oncology, National Cancer Institute, Naples, Italy; Virginia Commonwealth University, United States of America

## Abstract

**Background:**

Cancer Stem Cells (CSCs) hypothesis asserts that only a small subset of cells within a tumour is capable of both tumour initiation and sustainment. The Epithelial-Mesenchymal Transition (EMT) is an embryonic developmental program that is often activated during cancer invasion and metastasis. The aim of this study is to shed light on the relationship between EMT and CSCs by using LC31 lung cancer primary cell line.

**Materials and Methods:**

A549 and LC31 cell lines were treated with 2 ng/ml TGFβ-1 for 30 days, and 80 days, respectively. To evaluate EMT, morphological changes were assessed by light microscopy, immunofluorescence and cytometry for following markers: cytokeratins, e-cadherin, CD326 (epithelial markers) and CD90, and vimentin (mesenchymal markers). Moreover, RT-PCR for Slug, Twist and β-catenin genes were performed. On TGFβ-1 treated and untreated LC31 cell lines, we performed stemness tests such as pneumospheres growth and stem markers expression such as Oct4, Nanog, Sox2, c-kit and CD133. Western Blot for CD133 and tumorigenicity assays using NOD/SCID mice were performed.

**Results:**

TGFβ-1 treated LC31 cell line lost its epithelial morphology assuming a fibroblast-like appearance. The same results were obtained for the A549 cell line (as control). Immunofluorescence and cytometry showed up-regulation of vimentin and CD90 and down-regulation of cytocheratin, e-cadherin and CD326 in TGFβ-1 treated LC31 and A549 cell lines. Slug, Twist and β-catenin m-RNA transcripts were up-regulated in TGFβ-1 treated LC31 cell line confirming EMT. This cell line showed also over-expression of Oct4, Nanog, Sox2 and CD133, all genes of stemness. In addition, in TGFβ-1 treated LC31 cell line, an increased pneumosphere-forming capacity and tumours-forming ability in NOD/SCID mice were detectable.

**Conclusions:**

The induction of EMT by TGFβ-1 exposure, in primary lung cancer cell line results in the acquisition of mesenchymal profile and in the expression of stem cell markers.

## Introduction

Two important hypothesis have been postulated in the genesis, formation, growth, and metastasis of epithelial cancer: the role of Cancer Stem Cells (CSCs) or Tumour Initiating Cells (TICs) and the involvement of the so called Epithelial-Mesenchymal Transition (EMT).

Cancer stem cells have been defined as “a cell within a tumour that possess the capacity to self-renew and to cause the heterogeneous lineages of cancer cells that comprise the tumour” [1]. These two definitive biological properties are what make the CSCs the prime candidate for initiation of relapse.

CSCs hypothesis asserts that only a small subset of cells within a tumour is able of both tumour initiation and sustainment [2], [3]. These cells express stemness markers, are able to form floating spheres in serum-free medium, a property associated with stem cells, and are also able to differentiate in an aberrant cell phenotype constituting tumour heterogeneity [4]. Experimentally, this population is identified by its ability to form new tumours through serial transplantations in immunodeficient non-obese diabetic (NOD)/severe combined immunodeficient (SCID) mice, re-establishing tumour heterogeneity [5].

There are two basic topics that underline the hypothesis that CSCs originate from normal tissue stem cells. First of all, the CSCs have normal stem cell properties such as self-renewal, differentiation, drug resistance and migration capacity. Then, the longevity of stem cells make them susceptible to accumulating genetic and epigenetic damages so as to make them good candidates for the emergence of neoplastic transformation [6], [7]. The CSCs are the only cells that are capable of generating tumours similar to the original patient specimens when transplanted into immunocompromised mice such as NOD/SCID mice [8].

Existing therapies have enhanced the length of survival after diagnosis of cancer, but completely failed in terms of recovery. Cancer therapy failures may be due to inefficient effects of current therapy upon potentially quiescent CSCs, which remain vital and retain the capacity to regenerate the tumour [9]. In most cases, current therapeutic strategies are developed to target the bulk of cancer and likely do not eradicate CSCs completely. CSCs are more resistant to therapies, due to survival advantage with increased anti-apoptotic activities and drug resistance due to increased levels of drug efflux pumps such as BCRP (breast cancer resistance protein) and MDR (multi-drug resistance) complexes [10], [11].

The CSCs have been identified in a variety of solid tumours including glioblastomas [12], breast [13], and lung cancer [14], [15]. There are three distinct main methodologies to isolate CSCs from solid tumours: i) isolation of CSCs by flow cytometry according to CSC-specific cell surface markers such as CD44 or CD133; ii) detection of side population phenotype by Hoechst33342 exclusion; iii) the sphere formation under the cultivation of defined serum-free medium with growth factors which maintains the CSCs undifferentiated.

The Epithelial-Mesenchymal Transition (EMT) is an embryonic key developmental program that is often activated during cancer invasion and metastasis [16], [17]. It is a process by which cells undergo a morphological switch from the epithelial polarized phenotype to the mesenchymal fibroblastoid phenotype. As a result of EMT, epithelial cells lose their defined cell–cell/cell–substratum contacts and their structural/functional polarity, and they become spindle shaped and morphologically similar to activated fibroblasts [18]. At the molecular level, EMT is defined by the loss of cell–cell adhesion molecules (eg, E-cadherin and ZO-1), down-regulation of epithelial differentiation markers including cytokeratins and E-cadherin and transcriptional induction of mesenchymal markers such as vimentin, fibronectin and N-cadherin with a nuclear localization of beta-catenin [19]. Nuclear beta-catenin induces a gene expression pattern favouring tumour invasion, and mounting evidence indicates multiple reciprocal interactions of E-cadherin and beta-catenin with EMT-inducing transcriptional repressors to stabilize an invasive mesenchymal phenotype of epithelial tumour cells [20], [21]. Other genes involved in EMT are Snail, Twist e SIP-1/ZEB-2, all repressors of gene CDH1 that codes for E-cadherin [16]. Several distinct traits have been conveyed by EMT, including cell motility, invasiveness, resistance to apoptosis, and some properties of stem cells. Many signalling pathways have contributed to the induction of EMT, including transforming growth factor-beta (TGFβ-1), Wnt, Hedgehog, Notch, and nuclear factor-kappa B (NFkB) [22]. Kyoung-Ok Hong et al. [23] have shown that activation of PI3K/Akt axis is one of the key mechanisms in the process of EMT and it seems that its inhibition by treatment with phosphatidylinositol ether lipid analogues (PIA) may regulate the reverse process Mesenchymal Epithelial reverse Transition (MErT) leading to the re-expression of both E-cadherin and β-catenin, and reducing expression of vimentin, mesenchymal marker, in oral squamous lines carcinoma stabilized. During the process of tumour metastasis, which is often enabled by EMTs, disseminated cancer cells would seem to require self-renewal capability, similar to that exhibited by stem cells, in order to spread macroscopic metastases [24]. This raises the possibility that the EMT process, which enables cancer cell dissemination, may also impart a self-renewal capability to disseminating cancer cells. Indeed, the metastatic process is at least superficially similar to the processes that occur during tissue repair and regeneration and enable adult stem cells to exit tissue reservoirs such as the bone marrow, enter and survive in the circulation, and get into secondary tissue sites, where they proliferate, differentiate, and participate in tissue reconstruction [25]. Together, these diverse lines of evidence suggest a possible link between cancer stem cells and the mesenchymal-appearing cells generated by EMTs and the reverse process termed Mesenchymal Epithelial reverse Transition (MErT). Mani et colleagues [26] were the first to demonstrate such correlation in immortalized human mammary epithelial cells (HMLEs).

In this context, it is important to identify which factors could induce EMT and how the EMT cells could become a resource for cancer stem-like cells, developing novel and targeted therapies for lung cancer. Therefore, the aim of this study is to shed light on the possible relationship between EMT and CSCs by using LC31 primary cell line obtained from tissue sample after surgery in patient affected by Non Small Cell Lung Cancer (NSCLC).

## Results

### TGFβ-1 treatment induces morphologic changes in NSCLC cell lines

In order to investigate the effect of TGFβ-1 on LC31 and A549 cell lines, we treated them with 2 ng/ml of TGFβ-1. As already also demonstrated by Ju Hee Kim [27], A549 cells treated with TGFβ-1 lost their epithelial morphology observable after 48 hours of treatment; they were dispersed and assumed a fibroblast-like appearance with longed shape and central nucleus.

**Figure 1:**
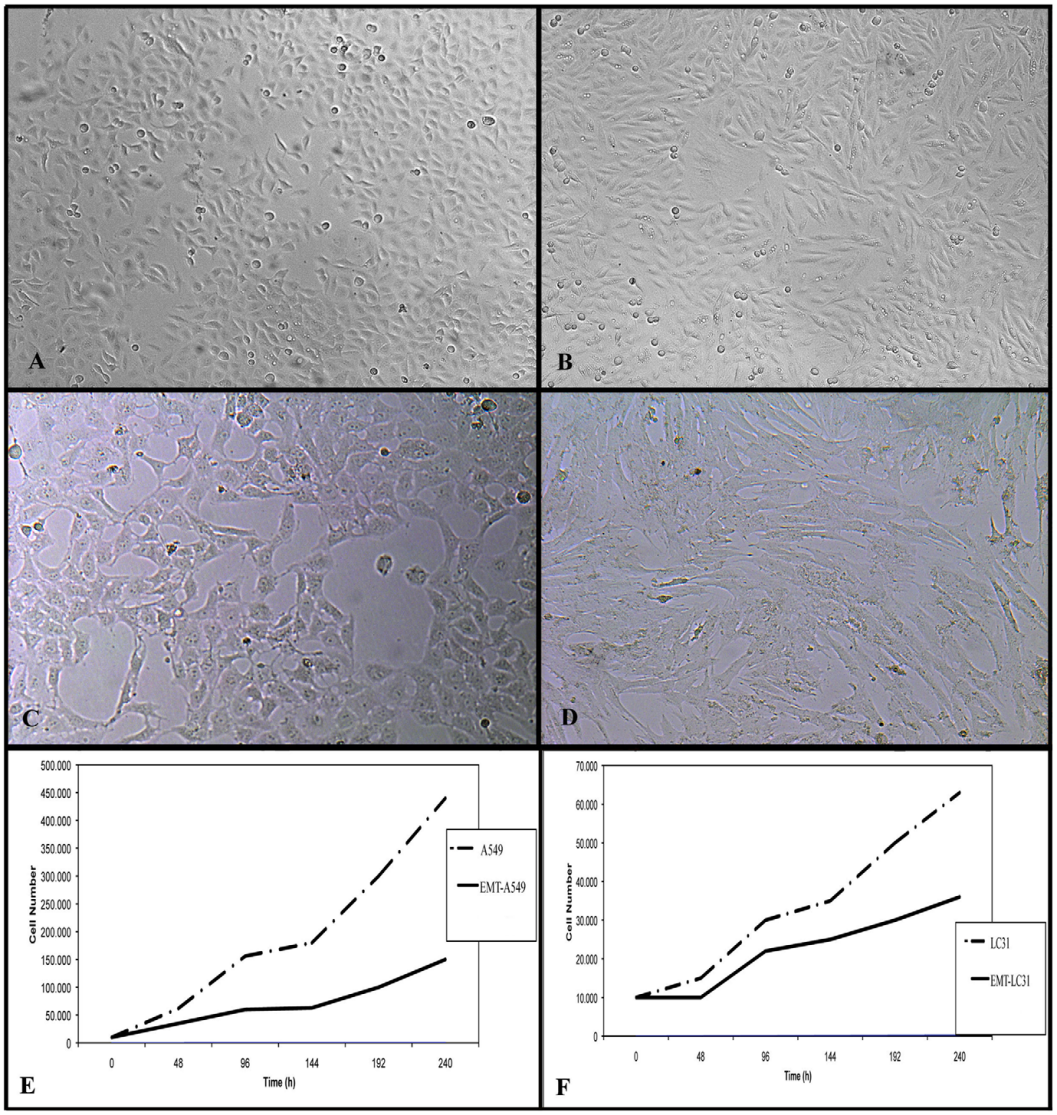
Morphological changes and growth curves after TGF-β1 treatment. A: untreated A549, OM 200×; B: treated A549, at 2 days TGFβ-1 treatment, OM 400×; C: untreated LC31, OM 400×; D: treated LC31, at 30 days TGFβ-1 treatment, OM 400×; E: growth curves of untreated and treated A549; F: growth curves of untreated and treated LC31.

LC31 cells treated with TGFβ-1 lost their epithelial morphology and acquired mesenchymal traits starting from 21 days of treatment. The cells became longed, fibroblast like with central nucleus and started to grow as bundles. This morphology was maintained for all time of treatment (Fig. 1A–D).

### TGFβ-1 treatment induces growth inhibition in NSCLC cell lines

In all cell lines tested, TGFβ-1 induced growth inhibition. In A549, growth curves analyses showed a strong growth inhibition during culture time with DT 28 h for treated A549 respect to DT 18 h of the corresponding untreated cell line. In LC31 cells, the DT was 72 h for treated cells whereas DT was 36 h for untreated cells (Fig. 1E–F).

### TGFβ-1 treatment promotes a shift from epithelial to mesenchymal phenotype

The morphological effect of TGFβ-1 on A549 and LC31 cell lines suggested that TGFβ-1 promoted an EMT. The morphological changes characteristic of cells undergoing EMT is accompanied by a shift in expression from an epithelial to a mesenchymal repertoire. To determine whether TGFβ-1 induced such shift, we used cytometry, immunofluorescence and RT-PCR to examine the expression and distribution of CD90, CD326, vimentin, e-cadherin and cytockeratins markers and β-catenin, Slug and Twist genes.

**Figure 2:**
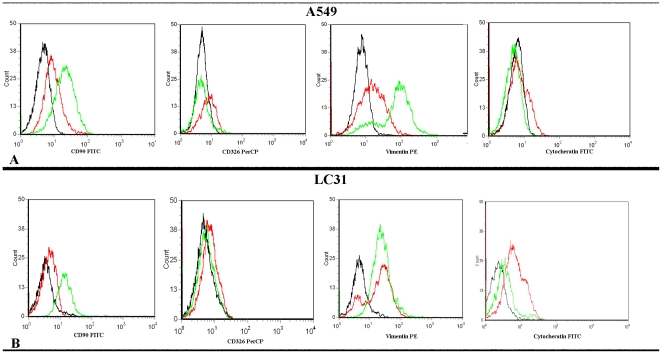
TGF-β1 up-regulates mesenchymal markers expression and down-regulates epithelial markers expression. A: Cytometric analysis for CD90, CD326, vimentin and Cytokeratin in untreated (line red) and TGFβ-1 treated [line green] in A549 cell line after 20 days of treatment; B: Cytometric analysis for CD90, CD326, vimentin and Cytokeratin in untreated [line red] and TGFβ-1 treated (line green) in LC31 cell line after 30 days of treatment. Isotype controls are in black.

According to cytometric analysis, in untreated cell lines, CD90 was weakly expressed on A549 (mean percentage 10%) and LC31 (mean percentage 4%). CD326 and cytokeratins expression levels were low both in A549 (mean percentage 10% and 1,1% respectively) and LC31 (mean percentage 1% and 18,6% respectively). Vimentin was weakly positive in A549 (mean percentage 22%) and LC31 (mean percentage 17%). After TGFβ-1 treatment, CD90 (mean percentage 93%) and vimentin (mean percentage 91%) levels increased, whereas CD326 and cytokeratins decreased in A549 and LC31 (Fig. 2A,B).

**Figure 3:**
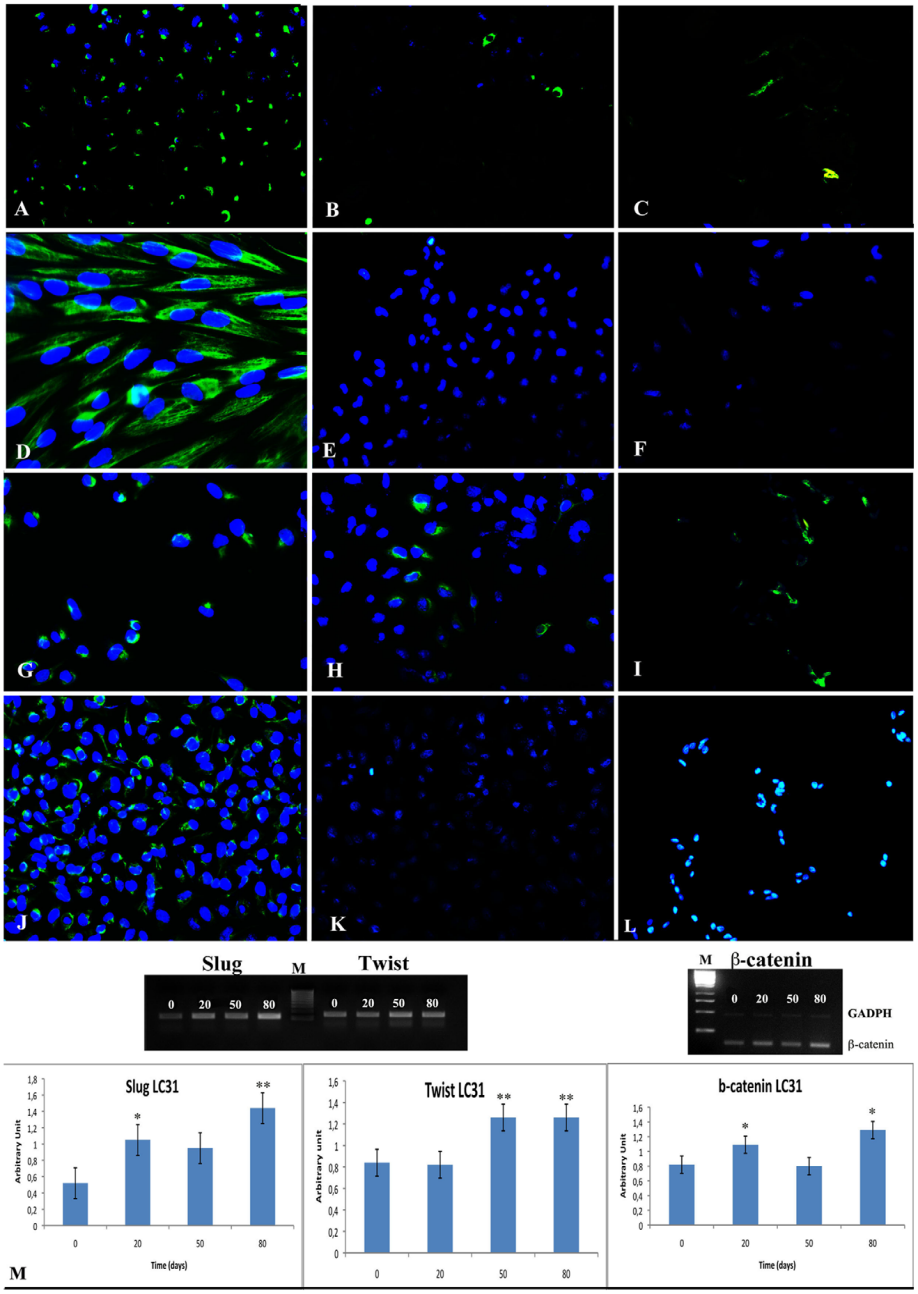
TGF-β1 promotes EMT. Immunofluorescence for vimentin (A), cytokeratin (B) and e-cadherin (C) on untreated A549 cell line; vimentin (D), cytokeratin (E) and e-cadherin (F) on treated in A549 cell line after 20 days of TGFβ-1 treatment; vimentin (G), cytokeratin (H) and e-cadherin (I) on untreated LC31 cell line; vimentin (J), cytokeratin (K) and e-cadherin (L) on treated in LC31 cell line after 30 days of TGFβ-1 treatment. All immunofluorescence images have OM 200×; M: RT-PCR analysis and densitometry evaluation for Slug, Twist and β-catenin on untreated and TGFβ-1 treated in LC31 cell line after 0, 20, 50 and 80 days of treatment. *, p<0,001, **, p<0,0001 compared to parental cell line (0 day of treatment).

In immunofluorescence assay, in the untreated cell lines, vimentin was expressed both in A549 and LC31 but it was contained in perinuclear vescicles. Cytokeratins and E-cadherin were weakly expressed in both cell lines. After TGFβ-1 treatment, the vimentin was strongly and uniformly distributed in all A549 and LC31 cells, whereas cytokeratins and E-cadherin remained weakly expressed and localized in perinuclear areas of cells until to be abrogated after 30 days for A549 and 40 days for LC31 (Fig. 3 A–L).

The RT-PCR data showed that there was massive shift of gene expression from a pattern characteristic of epithelial cells to that of mesenchymal cells in LC31 cell line with a considerable increase in the expression of EMT-inducing transcription factors, specifically beta-catenin (∼1,5 fold), Twist (∼1,5 fold), and Slug (∼2,7 fold) indicating their EMT phenotype. Up-regulation of these genes mentioned above was TGFβ-1 dependent time except for beta-catenin for which there was an increase at 20 and 80 days of treatment respect to untreated cell line and a weak decrease at 50 days respect 20 and 80 days of treatment but always increased respect to untreated cell line (Fig. 3M).

### Gene expression profiling of stemness markers in EMT-LC31 cell line

**Figure 4:**
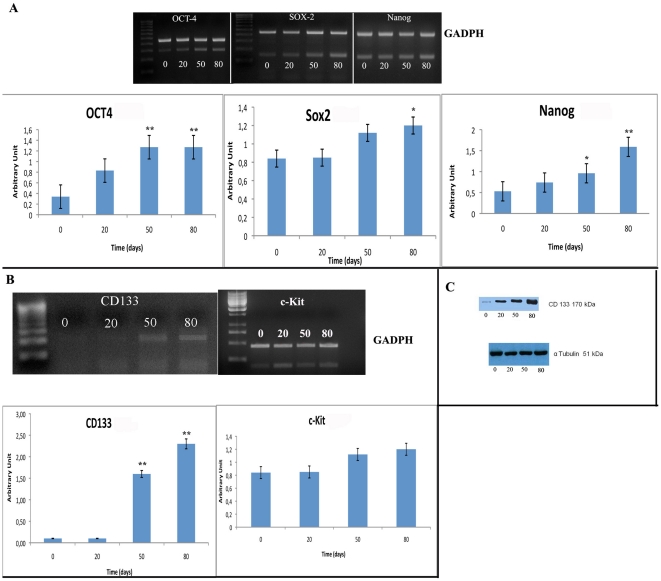
Stemness markers distribution on LC31 and EMT-LC31 cell lines. A: RT-PCR and densitometry evaluation for Oct4, Sox2 and Nanog genes on LC31 and EMT-LC31 cell lines after 0, 20, 50 and 80 days of treatment; B: RT-PCR and densitometry evaluation for CD133 and c-kit genes on LC31 and EMT-LC31 cell lines after 0, 20, 50 and 80 days of treatment; C: western blot for CD133 LC31 and EMT-LC31 cell lines after 0, 20, 50 and 80 days of treatment. *, p<0,001, **, p<0,0001 compared to LC31 cell line (0 day of treatment). α-tubulin is used as loading control.

To investigate the genes regulating and maintaining the stem cell phenotype of LC31 cells having EMT signatures (termed EMT-LC31 cell line), we performed RT-PCR for OCT4, Nanog and Sox2. Interestingly, transcription factors OCT4, Nanog, Sox2, known to be sufficient to reprogram mouse or human somatic cells to undifferentiated, pluripotent stem cells, were found to be significantly increased in EMT-LC31 cell line with an increase of 3,5 fold, 3,0 fold, and 1,4 fold for OCT4, Nanog and Sox-2, respectively more than parental cell line indicating their stemness phenotype (Fig. 4A).

### CD133 and c-kit markers were increased in EMT-LC31 cell line

To further determine whether cells with EMT phenotype could show cancer stem like cell characteristics, we evaluate expression of CD133, main marker in identifying cancer stem cells in NSCLC [14], [15] and c-kit [28], mesenchymal stem marker.

Cytometry analyses showed that in LC31 parental cell line, the percentage of CD133 was 3% of total cell population. After TGFβ-1 treatment, the results showed that no differences in CD133 expression levels between parental and EMT-cells were detectable (data not shown). This result is different respect to those obtained by RT-PCR and western blotting for CD133. Both RT-PCR and western blotting showed an increase of CD133 (∼2,3 fold for RT-PCR) after different times of TGFβ-1 treatment. Regarding c-Kit gene, the latter resulted up-regulated in EMT-LC31 cells with an increase of ∼2,3 fold more than parental cell line (Fig. 4B,C).

### Pneumospheres formation ability was increased in EMT-LC31 cell line

Liu et al. [29] and others [30] have demonstrated that the ability to form mammospheres *in vitro* depends on the presence of self-renewing. We tested pneumophere-forming ability of EMT-LC31 cells compared to parental cell line.

**Figure 5:**
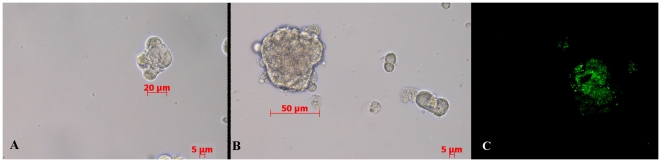
Pneumospheres formation ability evaluation and CD133 expression on LC31 and EMT-LC31 cell lines. A: LC31 pneumospheres; EMT-LC31 pneumospheres; C: CD133 expression on EMT-LC31 pneumospheres.

Significantly, after we induced an EMT in LC31 cells by exposing them to TGF-β1, we found that EMT-LC31 cells showed significantly increased ability to form pneumopheres compared to parental cell line forming at least >40-fold more pneumospheres than parental cells. The ratio of pneumospheres size and their growth was significantly greater than those of untreated cells. Moreover, the EMT pneumospheres were more than 50 µm in diameter after 5 days, while parental pneumospheres were 20 µm (Fig. 5A,B). In addition all EMT pneumospheres were positive for CD133 (Fig. 5C). Based on this functional assay, we concluded that the cells generated by an EMT acquired yet another attribute of lung cancer stem cells.

### Colony efficiency analyses

**Figure 6:**
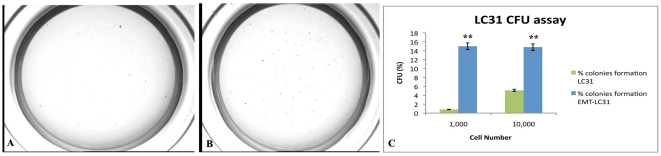
Colony efficiency analyses. A: photographs of colonies from LC31 cell line and [B] EMT-LC31 cell line; C: the colony number was counted and the data were presented as CFU (%) respect to cell number seeded.

One of the methods of analysing the tumorigenic potential is the soft agar assay that measures anchorage-independent growth, which is an indicator for cell transformation. As described in Table 1, assessment of growth kinetics revealed major colony efficiency of EMT-LC31 cell line compared to parental cell line with 18 and 3 fold increase for 1,000 and 10,000, respectively, of seeded EMT cells respect to parental cell line (Fig. 6A–C).

**Table 1:** % colonies formation for LC31 cell line.

N° seeded cells	% colonies formation LC31	% colonies formation EMT-LC31	Fold increase	p-value
1.000	0.82	15	18	0.0001
10.000	5.10	14.80	3	0.002

### EMT-LC31 generates tumours greater than corresponding parental cell line

In our previous studies [15], we showed that the tumours from LC31 cells grown as pneumopsheres grew much faster compared to that from corresponding cells grown in adherent culture condition. In order to test whether TGFβ-1 treatment succeeded in altering the tumour-initiating frequency of transformed cells, we injected EMT-LC31 cells and corresponding cell line into immunodeficient hosts. As reported in Table S1, we found that the volume of tumour induced by EMT-LC31 cells was significantly larger than that of parental cells (Fig. S1). These results suggest that the cells with EMT signature promoted tumorigenicity *in vivo*.

## Discussion

Epithelial to mesenchymal transition (EMT) is a fundamental physiologic process whereby epithelial cells lose their polarity and undergo a transition to a mesenchymal phenotype. Hallmarks of EMT include loss of cell-cell adhesion, re-organization of the cytoskeletal actin and acquisition of increased migratory characteristics [31], [32].

EMT is a crucial event in tumour progression and several studies state that the EMT is activated in many types of cancers [33]–[35]. Despite recent progress, further studies are needed to clarify the role of EMT in the invasion and metastasis of tumours. During the process of tumour metastasis, cancer cells, that metastasize, acquire skills of self-renewal, similar to that exhibited by stem cells in order to spread the metastases [24].

Together, these evidences suggest a possible link between cancer stem cells and the mesenchymal-appearing cells generated by EMTs.

In this context, aim of this study is to show that EMT acquisition is associated with an increase of stemness signatures in a primary cell line obtained in our laboratory. A549 lung cancer is a well-characterized cell line that has been used as a model system to study the mechanisms of carcinogenesis, apoptosis and cancer progression in lung cancer [36], [37]. Because EMT plays a key role in the tumor progression, we chose A549 cell line as model system of EMT. We focused our attention on LC31 cell line obtained in our laboratory and investigate the effect of TGFβ-1 on EMT and stemness mechanism on this line. In agreement with previous reports [38], [39], we showed that TGFβ-1 induces EMT in A549 cells by acquisition of mesenchymal morphology, increased expression of mesenchymal markers such as vimentin and CD90 and decreased expression of epithelial markers such as cytokeratins and CD326. Once defined that our EMT study model is useable, we tested the effect of TGFβ-1 on LC31 cell line. We treated LC31 cell line with TGFβ-1 2 ng/ml for 80 days. Only after about 20 days, we observe morphological changes that are consistent with the acquisition of EMT phenotype as characterized by the loss of expression of epithelial markers such as cytokeratins, e-cadherin and CD326 and the gain or increased expression of mesenchymal markers such as vimentin replacing cytokeratins. After 20 days of treatment, LC31 cells became elongated, central nucleus with fibroblast like shape and increased stress fiber reorganization.

EMT takes 48 hours in A549 because it has stronger mesenchymal markers expression than LC31 needing 20 days to undergo EMT.

Therefore, the LC31 cell line displays several features typical of EMT: reduction in cell-cell adhesion, flattening and scattering, expression of mesenchymal markers. Slug, Twist and β-catenin, main transcriptional factors involved in EMT, are up-regulated in the cells treated and these factors increase with increasing TGFβ-1 treatment time, confirming their EMT phenotype.

These results open the way to verify if LC31 primary lung cancer cell line, sensitive to TGFβ-1 treatment, could increase stem cell characteristics. Interestingly, EMT-LC31 cells also display stem-like cell phenotype characterized by increased pneumosphere-forming capacity compared with parental LC31 cell line with EMT pneumospheres greater than parental pneumopheres. Moreover EMT pneumospheres were positive for CD133 marker reinforcing the stemness signature. Most importantly, it is well known that the co-expression of Sox-2, Nanog and Oct4 in human or mouse somatic cells can reprogram these cells into pluripotent embryonic stem-like cells [40], [41]. In our study, we found that the expressions of Sox-2, Nanog and Oct4 were dramatically up-regulated in EMT-LC31 cell line compared to parental cell line. Taken together these data, LC31 cell line with EMT signature showed an increase of stem-like cell characteristics associated with over-expression of Sox-2, Nanog and Oct4.

Recent studies have shown that c-kit and its ligand are expressed in lung cancer. Immunohistological studies on the c-kit expression showed that the protein is aberrantly expressed only in lung cancer cells and not in pneumocytes or normal bronchial epithelial cells [42]. In addition, Levina et al. [43], [44] have showed that the treatment of lung tumour cells with doxorubicin, cisplatin, or etoposide resulted in the selection of drug surviving cells expressing CD133, CD117, SSEA-3, TRA1-81, Oct-4, and nuclear beta-catenin with low expression of the differentiation markers cytokeratins 8/18. In our study, we demonstrate that TGFβ-1 induces also an increase of c-kit m-RNA expression, another stemness marker, reinforcing the hypothesis that LC31 cell line with EMT signature showed an increase of stem phenotype. Another interesting result is the up-regulation of CD133 in EMT-LC31 cell line, main marker for CSCs identification in lung cancer as reported by Eramo et al. [14] and Tirino et al. [15]. In our study, we observed an increase of CD133 both in RT-PCR and Western blot, but not in cytometry and immunofluorescence on adherent cells. There are many works in which doubts about the limitations in the use of antibody are discussed [45]. The antibodies more commonly used recognize AC133 and AC141 highly glycosylated epitopes [46]. Therefore, their use carries the risk of underestimated non glycosylated forms of the antigen. Moreover, several CD133 mRNA splicing have been identified, which would in turn give rise to protein products not recognizable by common antibodies; finally, intrinsic limitations of immunofluorescence and cytometry methodologies, which implies antibody sensitivity, epitope damage due to routine fixation/inclusion procedures, antigen retrieval, enzymatic steps of cell preparation must be taken into account [47]. In this context, we can also hypothesize that TGFβ-1 treatment give rise to conformational change of CD133 epitopes and its antibody is not able to recognize the molecule in cytometry and immunofluorescence procedures. In the pneumospheres, most probably, tridimensional structure allows the exposure of the epitopes recognized by common antibodies. Moreover, they are not subject to enzymatic steps that could damage cellular structure.

Although this consideration, RT-PCR and Western blot confirm the same the stemness feature of EMT-LC31 cell line after treatment of TGFβ-1 with up-regulation both CD133 m-RNA and CD133 protein.

Finally, to evaluate TGFβ-1 effect on tumorigenic potential , we performed both soft agar assay and tumorigenicity *in vivo*. TGFβ-1, in line with cancer stem cells concept, induces an increase of colonies formation in soft agar experiments and the volume of tumours was significantly larger than that of untreated LC31 cell line confirming that EMT phenotype not only promoted stemness phenotype but also the tumorigenicity. In conclusion, the cells with EMT phenotype promoted tumorigenicity.

Therefore, the high probability that the EMT generates cells with many of the properties of self-renewing stem cells is verified by increased expression of Nanog, Oct4 and Sox2 , determinant self-renewal factors and CD133 and c-Kit, main stemness markers of CSCs in lung cancer.

In conclusion, we report that the induction of EMT by TGFβ-1 treatment in a primary lung cancer cell line results in the acquisition of mesenchymal profile and in up-regulation of the expression of stem cell markers.

This study highlights the possibility to address a novel pharmacological approach versus EMT-phenotype cells or cancer stem-like cells for the prevention of tumour progression and metastasis formation.

## Materials and Methods

### Ethics Statement

The experimental protocols have been evaluated and approved from the ethical committee on animal use of Biogem. The experimental project have the approval ID 10.08 and have been communicated in May 26^th^, 2008 to the Italian Minister of Health, following the national laws concerning the protection of animal welfare. The patient, enrolled in this study, had signed informed consent, approved by our Internal Ethical Committee (National Cancer Institute, Naples).

### Cell Culture

A549 cell line was purchased from ATCC Cell Bank and was cultured in RPMI1640 (Lonza, Milan, Italy) at 10% fetal bovine serum (FBS) at 37°C, 5%CO_2_. LC31 cell line has been obtained in our laboratory by a patient affected from lung squamous adenocarcinoma with written informed consent, approved by our Internal Ethical Committee (National Cancer Institute, Naples) and was cultured in IMDM (Lonza) at 10% FBS [14]. For experiments, cells were grown to 90% confluence.

### TGFβ-1 treatment and Growth Curves

In order to induce EMT process, A549 and LC31 cell lines were treated with 2 ng/ml TGFβ-1 (AbCAM, Milan, Italy) for 30 and 80 days, respectively. TGFβ-1 has been added twice a week in the medium. To test the possible growth inhibition due to TGFβ-1 treatment, 10,000 cells were plated in 24well plates for each cell line and TGFβ-1 untreated and treated cells were detached every 24 hours for 10 days. The number of cells for each experimental condition was counted and represented on a linear graph. The doubling time (DT) was determined from the growth curves by using the formula:
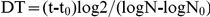
where t and t_0_ were the times at which the cells were counted, and N and N_0_ were the cell numbers at times t and t_0_, respectively.

### Pneumospheres assay

In order to evaluate the effect of TGFβ-1 on pneumospheres growth and formation, the LC31 cell line was plated at a density of 60,000 cells per well in six-well ultra-low attachment plates (Corning Inc., Corning, NY, USA) in BEBM cell medium, supplemented with BEGM [prepackaged SingleQuots containing retinoic acid, bovine pituitary extract, insulin, hydrocortisone, transferrin, triiodotyronine, epinephrine, human epidermal growth factor, gentamicin and amphotericin B (all from Lonza Group Ltd., Basel, Switzerland) plus human EGF (10 ng/ml; Sigma, Milan, Italy) and human bFGF (10 ng/ml; Sigma, Milan, Italy). Cells were incubated in a humidified atmosphere at 37°C with 5% CO_2_. Fresh aliquots of TGFβ-1, EGF and bFGF were added twice a week. After 48–72 h of culture, spheres were visible at inverted phase-contrast microscopy.

### Flow Cytometry

In order to evaluate the effect of TGFβ-1 on both stemness and differentiation phenotype of A549, and LC31 cells lines, 200,000 cells were stained with the following antibodies (2 µg/ml): mouse anti-human CD90 FITC (Becton & Dickinson, Buccinasco, Milan, Italy), mesenchymal marker, mouse anti-human CD133 PE (Miltenyi Biotec, Calderara di Reno, Bologna, Italy), mouse anti-human CD326 PerCP (EpCAM, Becton & Dickinson), epithelial marker, mouse anti-human vimentin (DAKO, Milan, Italy) and mouse anti-Cytokeratin (clone CK3-6H5, Miltenyi Biotec). The antibodies were incubated for 30 minutes at 4°C in the dark. After incubation, the samples were washed with PBS and analyzed by FACSAriaII (Becton Dickinson). For vimentin and citokeratin intracellular staining, Fix and Perm kit (Invitrogen, Milan, Italy) was used according manufacturer's instructions. The secondary antibody for vimentin assay was goat anti-mouse PE conjugated (AbCAM). All data were analysed by Diva Software.

### Immunofluorescence Assay

TGFβ-1 untreated and treated A549 and LC31 cells were plated in 24 well plates and were fixed with 70% ethanol/0,1% Triton for 30 minutes at 4°C, washed with PBS, treated with 5% Bovine Serum Albumin for 60 minutes at room temperature and then stained with primary antibodies at 4°C over night. The primary antibodies used were mouse anti-human CD133/1 (Miltenyi Biotec), mouse anti-human vimentin (DAKO), anti-human E-cadherin (DAKO) and mouse anti-human citokeratin (DAKO). The secondary antibody, goat anti-mouse FITC (AbCAM) diluted 1∶200 in PBS, was incubated for 60 min at 4°C, and the DAPI (Sigma, Milan, Italy), used to stain the nucleus, was incubated for 7 minutes at room temperature. The same procedure for CD133 staining was performed on LC31 cells grown as pneumospheres after treatment of TGFβ-1. Cells were then washed twice as described above and observed under the fluorescence microscope (Zeiss, Milan, Italy). Isotypes and non-probed cells were used as controls.

### RT-PCR

Total RNA was extracted using TRIzol Reagent (Invitrogen, Milan, Italy) according to the manufacturer's protocol. RNA concentration and purity were determined by A_260_ and A_260_/A_280_ ratios, respectively. The integrity of total RNA was assessed on standard 1% agarose/formaldehyde gels. The RNA samples were treated with DNase I to remove residual traces of DNA. cDNA was obtained from 1 µg of total RNA, using reverse transcriptase (Promega Italia Srl, Milan, Italy) and random primers (Promega) in a final volume of 20 µl. cDNAs (1 µl for each sample) were amplified by PCR using the primer sequences as follows:

**CD133:** 5′-TCTTGACCGACTGAGACCCAAC-3′(sense) and 5′-ACTTGATGGATGC ACCAAGCAC-3′(antisense); **OCT3/4**: 5′-ACATGTGTA AGCTGCGGCC-3′ (sense) and 5′-GTTGTGCATAGTCGCTGCTTG -3′ (antisense); **Nanog:** 5′-TTCAGTCTGGACACTGGCTG-3′ (sense) and 5′-CTCGCTGATTAGGCTCCAAC-3′ (antisense); **SOX2**: 5′-CGATGCCGACAAGAAAACTT-3′ (sense) and 5′-CAAACTTCCTGCAAAGCTCC-3′ (antisense); **Twist**: 5′-TCTCGGTCTGGAGGATGGAG-3′(sense) and 5′-GTTATCCAGCTCCAGAGTCT-3′ (antisense); **Slug**: 5′-GAGCATTTGCAGACAGGTCA-3′ (sense) and 5′-CCTCATGTTTGTGCAGGAGA -3′ (antisense); β- catenin: 5′-GCCGGCTATTGTAGAAGCTG-3′ (sense) and 5′-GAGTCCCAAGGAGACCTTCC-3′ (antisense); **c-kit:** 5′-CCGGTCGATTCTAAGTTCTAC-3′ (sense) and 5′-GATTGGTGCTCTCTGAAATCTG-3′ (antisense).

Thermal cycle parameters were: 95°C for 2 minutes, 35 cycles of 95°C for 30 seconds, 52–60°C (depending on the T_m_ of each individual set of primers) for 1 minute and 72° for 30 seconds. **GAPDH**: 5′-TGGACTCCACGACGTACTCAG-3′ (sense) and 5′-ACATGTTCC AATATGATTCCA-3′ (antisense) was amplified as an internal control. The RT-PCR products were separated by 2% agarose gel electrophoresis, stained with ethidium bromide, and photographed under UV illumination. RT-PCR was performed on TGFβ-1 treated and untreated LC31 cell line at different treatment times (0, 20, 50 and 80 days).

### Western Blotting

Total cell lysates of TGFβ-1 treated and untreated LC31 cell line at different treatment times [0, 20, 50 and 80 days] were obtained by lysing the cells in RIPA buffer containing 50 mM Tris-HCl, 150 mM NaCl, 1% NP-40, 0,1% SDS, 0,5% sodium deoxycholate, 2 mM sodium fluoride, 2 mM Na_3_VO4_2_, 1 mM EDTA, 1 mM EGTA and protease inhibitor cocktail. Protein concentration was determined using bicinchoninic acid protein assay (Pierce, Rockford, IL). The proteins were separated by SDS-PAGE, transferred to nitrocellulose, blocked, and incubated with the following primary antibodies: CD133 (AbCAM) diluted 1∶500 and α-tubulin (Sigma, Milan, Italy) diluted 1∶100 that was used as loading control. The membrane was washed and incubated with the respective secondary antibodies conjugated with peroxidase. Protein detection was done with chemiluminescence detection system (Pierce).

### Soft Agar Assay

To evaluate the clonogenicity due to TGFβ-1 treatment, treated and untreated LC31 cells, at a density of 1,000, 5,000 and 10,000 cells per well in 24-well plates were plated in soft agar , in triplicate. The test was performed using 0.8% and 0.3% agar in IMDM as the base and top layers, respectively.

Cells were incubated for 21 days at 37°C in a humidified atmosphere at 5% CO_2_ in air and 50 µl of IMDM culture medium were added twice a week. At the end of the incubation period, colonies were stained with nitrobluetetraziolium (NBT, Sigma, Milan, Italy) at a concentration of 50 mg/100 ml in PBS and counted using an inverted microscope (Nikon TS 100, Milan, Italy). The colony efficiency was calculated as proportion of colonies per total number of seeded cells. The data were analyzed by Image Pro Plus software.

### Nonobese diabetic (NOD)/severe combined immunodeficiency (SCID) xenotransplantation

In order to evaluate the effect of TGFβ-1 on tumorigenicity, *in vivo* experiments were performed. TGFβ-1 untreated and treated LC31 cells grown as pneumospheres were subcutaneously injected in NOD/SCID mice (Charles River, Wilmington, MA). For this purpose, cells were harvested diluted in PBS, mixed with matrigel and injected subcutaneously in six-week-old female NOD/SCID mice at following serial dilutions: 1 and 5×10^4^; 1 and 5×10^5^; 1×10^6^ cells. After 60 days, mice were sacrificed and the tumour tissue collected, in part fixed in buffered formalin and subsequently analysed by immunohistochemistry and in part minced to re-obtain the cell line. Haematoxylin and eosin staining followed by immunohistochemical analysis were performed to analyse tumour histology. The injection experiments were in triplicate.

### Statistical analyses

The data are presented as the mean values ± SD. Comparison between groups were evaluated by a two-tailed student's t test. Values of p<0,05 were considered statistically significant.

## Supporting Information

Figure S1 **EMT-LC31 cells promoted tumour growth.** A: Hematoxylin and Eosin evaluation of the LC31 human tumour; B: Hematoxylin and Eosin evaluation starting from EMT-LC31 cells injected in NOD/SCID mice that resembles human original tumour; C: Tumor growth curve showing EMT-LC31 cells promote tumor growth in NOD/SCID mice much faster than LC31 cells starting from 100,000 cells injected.
(TIF)

Table S1 Tumour incidence of LC31 cells versus EMT-LC31 cells injected in NOD/SCID mice in limiting dilutions.
(DOC)
