# Supplementary figures and images for: Epithelial to Mesenchymal Transition by TGFβ-1 Induction Increases Stemness Characteristics in Primary Non Small Cell Lung Cancer Cell Line
Source: PLoS One. 2011 Jun 30;6(6):e21548. doi: 10.1371/journal.pone.0021548 (PMC3128060; doi:10.1371/journal.pone.0021548)

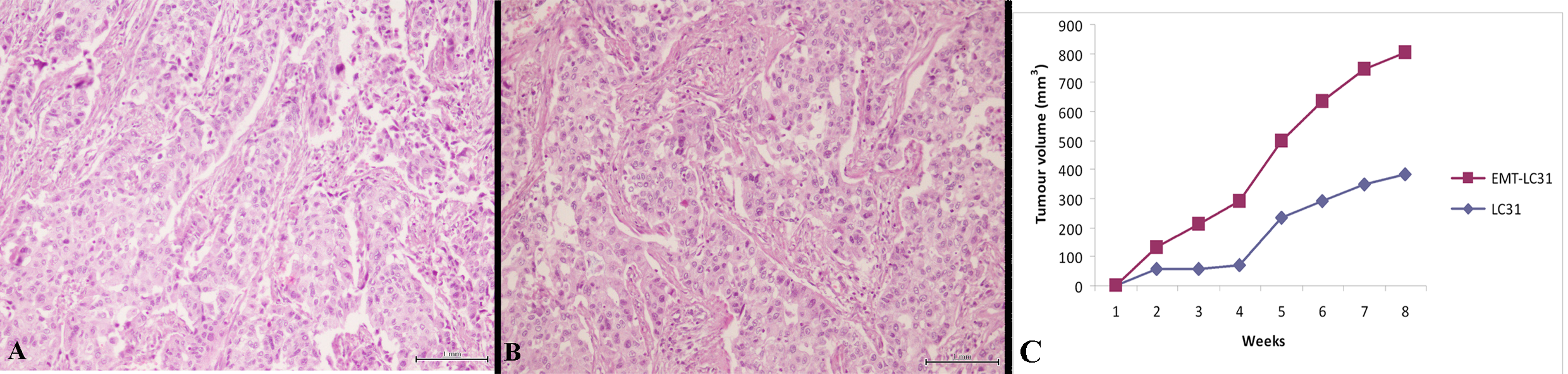

Supplement: Figure S1 — EMT-LC31 cells promoted tumour growth. A: Hematoxylin and Eosin evaluation of the LC31 human tumour; B: Hematoxylin and Eosin evaluation starting from EMT-LC31 cells injected in NOD/SCID mice that resembles human original tumour; C: Tumor growth curve showing EMT-LC31 cells promote tumor growth in NOD/SCID mice much faster than LC31 cells starting from 100,000 cells injected. (TIF) [file pone.0021548.s001.tif]
