# Supplementary material for: Epithelial to Mesenchymal Transition by TGFβ-1 Induction Increases Stemness Characteristics in Primary Non Small Cell Lung Cancer Cell Line
Source: PLoS One. 2011 Jun 30;6(6):e21548. doi: 10.1371/journal.pone.0021548 (PMC3128060; doi:10.1371/journal.pone.0021548)
Supplement: Supplementary file 2 [file pone.0021548.s002.doc]

Table S1. Tumour incidence of LC31 cells versus EMT-LC31 cells injected in NOD/SCID mice in limiting

	Tumors Incidence/Number of Injections				
Cells Injected	$1x10^6$	5×10^5	1×10^5	$5x10^4$	$1x10^4$
LC31	4/6	4/6	3/6	2/6	2/6
EMT-LC31	6/6	6/6	5/6	5/6	4/6
